# Core‐Shell Si@SiOC Particles Synthesized Using Supercritical Carbon Dioxide Fluid for Superior Li‐Ion Storage Performance

**DOI:** 10.1002/advs.202401350

**Published:** 2024-06-17

**Authors:** Rahmandhika Firdauzha Hary Hernandha, Bharath Umesh, Jagabandhu Patra, Chun‐Yen Chen, Ju Li, Jeng‐Kuei Chang

**Affiliations:** ^1^ Department of Materials Science and Engineering National Yang Ming Chiao Tung University 1001 University Road Hsinchu 30010 Taiwan; ^2^ Hierarchical Green‐Energy Materials (Hi‐GEM) Research Center National Cheng Kung University 1 University Road Tainan 70101 Taiwan; ^3^ Department of Nuclear Science and Engineering and Department of Materials Science and Engineering Massachusetts Institute of Technology 77 Massachusetts Avenue Cambridge MA 02139 USA; ^4^ Department of Chemical Engineering Chung Yuan Christian University 200 Chung Pei Road Taoyuan 32023 Taiwan

**Keywords:** green process, high energy density, high‐stability anode, silicon oxycarbide, supercritical fluid

## Abstract

A supercritical carbon dioxide (SCCO_2_) fluid, characterized by gas‐like diffusivity, near‐zero surface tension, and excellent mass transfer properties, is used as a precursor to produce silicon oxycarbide (SiOC) coating. SCCO_2_ disperses and reacts with Si particles to form an interfacial layer consisting of Si, O, and C. After an 850 °C annealing process, a conformal SiOC coating layer forms, resulting in core‐shell Si@SiOC particles. High‐resolution transmission electron microscopy and its X‐ray line‐scan spectroscopy, X‐ray photoelectron spectroscopy, Fourier‐transform infrared spectroscopy, and Raman spectroscopy, are used to examine the SiOC formation mechanism. Effects of SCCO_2_ interaction time on the SiOC properties are investigated. The SiOC layer connects the Si@SiOC particles, improving electron and Li^+^ transport. Cyclic voltammetry, galvanostatic intermittent titration technique, and electrochemical impedance spectroscopy are employed to examine the role of SiOC during charging/discharging. Operando X‐ray diffraction data reveal that the SiOC coating reduces crystal size of the formed Li_15_Si_4_ and increases its formation/elimination reversibility during cycling. The Si@SiOC electrode shows a capacitiy of 2250 mAh g^−1^ at 0.2 A g^−1^. After 500 cycles, the capacity retention is 72% with Coulombic efficiency above 99.8%. A full cell consisting of Si@SiOC anode and LiNi_0.8_Co_0.1_Mn_0.1_O_2_ cathode is constructed, and its performance is evaluated.

## Graphical abstract

A unique supercritical carbon dioxide method to create core‐shell Si@SiOC composite particles is developed. The conducting SiOC layer brings the particles closer together, benefiting electron and Li^+^ transport. In addition, the buffering SiOC layer can accommodate the Si volume change during lithiation/delithiation. The obtained Si@SiOC anode material has great potential for high‐energy‐density and high‐reliability battery applications.

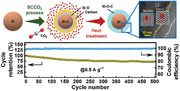

## 1 Introduction

Increasing demand for mobile electronic products, electric vehicles, and large‐scale energy storage systems has stimulated the improvements in Li‐ion battery (LIB) technology. Because of the large quantity of electrical power needed for various applications, high energy density is a major focus of LIB development.^[^ ^1^ ^]^ However, conventional graphite negative electrodes have limited capacity (372 mAh g^−1^), and thus a higher‐capacity electroactive material is required.^[^ ^2^ ^]^ In this context, Si‐based negative electrodes are of great interest. In addition to its abundance (28% of Earth's crust by mass), low cost, and nontoxicity, Si has a high theoretical capacity of ≈3579 mAh g^−1^ and a desirable lithiation/delithiation potential.^[^ ^3^ ^]^ Nevertheless, Si electrodes usually suffer from large volume expansion/contraction during cycling, which leads to electrode pulverization/collapse,^[^ ^4^ ^]^ repeated solid‐electrolyte interphase (SEI) breakdown/reformation, electrolyte consumption, and cyclable Li^+^ loss, which limit the electrode lifespan.^[^ ^5^ ^]^ Numerous Si nanostructures have been developed to release the induced stress and overcome the above problems.^[^ ^6^, ^7^ ^]^ However, these nanostructures decrease the electrode volumetric density and lower the initial Coulombic efficiency (CE). The preservation of some empty buffer space within the electrode to accommodate the Si volume change has also been proposed.^[^ ^8^, ^9^ ^]^ However, the voids introduced reduce the Si mass loading and, thus, the electrode energy density, making this approach impractical. An attractive alternative approach is to apply a surface carbon coating onto Si. Unfortunately, coated carbon materials have low specific capacities and can be brittle.^[^ ^10^, ^11^ ^]^ A more effective coating/buffering material is thus needed to improve Si electrode cyclability.

Silicon oxycarbide (SiOC) typically has an amorphous structure with mixed Si, C, and O atoms, and its chemical composition can be generally described as SiO_2(1‐_ *_x_*_)_C*_x_* + *y* C_free_.^[^ ^12^, ^13^ ^]^ Basically, SiOC is composed of Si/O/C repeated tetrahedral units as a primary framework, a free carbon phase, and some voids.^[^ ^14^ ^]^ In a tetrahedral unit, silicon is simultaneously bonded with carbon and oxygen and can be denoted as [C*_x_* SiO_4‐_ *_x_*], where *x* = 1, 2, or 3.^[^ ^15^ ^]^ The incorporated carbon, which can be four‐coordinated, in the silicate structure partially replaces some oxygen, which is two‐coordinated.^[^ ^16^ ^]^ This increased bonding number per anion can strengthen the molecular structure of the glass network, leading to enhanced thermal and mechanical properties.^[^ ^16^, ^17^ ^]^ This primary network provides an adequate Li^+^ storage capacity^[^ ^18^ ^]^ (i.e., the Li^+^ ions can alloy with Si atoms, bond with O atoms, and stay in the voids near C atoms^[^ ^19^, ^20^ ^]^ and a unique flexible characteristic).^[^ ^21^ ^]^ The latter feature is especially important for Si‐based anodes for LIBs. A resilient material is essential for reducing the damage associated with the significant volume change during cycling. In addition, the free carbon phase and voids in SiOC provide conductivity and a buffer zone for volume expansion, respectively, both of which are beneficial for electrode cyclability.^[^ ^14^, ^22^ ^]^

Some studies have attempted to incorporate SiOC into Si‐based anodes.^[^ ^23^, ^24^, ^25^, ^26^ ^]^ Kaspar et al. separately mixed nano‐crystalline and nano‐amorphous Si particles with polyorganosiloxane in acetone.^[^ ^23^ ^]^ After pyrolysis at 1100 °C under Ar, SiOC/Si_crystalline_ and SiOC/Si_amorphous_ composites were obtained. The former electrode had an initial capacity of ≈800 mAh g^−1^ and a CE value of 75% and the latter electrode had an initial capacity of ≈600 mAh g^−1^ and a CE value of 63%. Choi et al. developed an aerosol‐assisted chemical vapor deposition process using phenyltriethoxysilane solution,^[^ ^24^ ^]^ After a heat treatment at 800 °C in an Ar atmosphere, an SiOC glass coating was created on Si particles. An electrode based on these particles had an initial capacity of 2093 mAh g^−1^ (with an initial CE of 72%). In another work, poly‐phenylsilsesquioxane nanospheres were used to produce an SiOC skeleton (via pyrolysis), which served as both a mechanically robust buffer to accommodate the volume expansion of Si and an effective electron conductor in the electrode.^[^ ^25^ ^]^ The obtained Si/SiOC composite exhibited reversible capacities of ≈800 and ≈600 mAh g^−1^ at current densities of 100 and 500 mA g^−1^, respectively. With the addition of cetrimonium bromide as a surfactant during the interaction between silicone oil and Si nanoparticles, Jang et al. fabricated an Si/SiOC composite.^[^ ^26^ ^]^ The obtained anode material exhibited a good reversible capacity of 1649 mAh g^−1^ (with an initial CE of ≈78%) and decent cycling stability (1312 mAh g^−1^ at the 100th cycle at 0.5 A g^−1^). These studies show that the synthesis of SiOC is complex and usually requires the use of various solvents, silicone oil, siloxane, silane, and other Si/O/C‐containing polymeric‐based precursors.^[^ ^23^, ^24^, ^25^, ^26^ ^]^ This leads to a high environmental impact and a complicated process that is both costly and time‐consuming. In addition, most of the previous syntheses using polymer precursors resulted in SiOC particles with a large size and relatively low uniformity.^[^ ^27^, ^28^ ^]^ Furthermore, due to the amorphous nature of SiOC, which allows high freedom in chemical composition, the preparation method can affect the physicochemical properties and electrochemical performance of SiOC. Our goal is to develop a green, effective, and scalable method for applying an appropriate SiOC coating onto individual Si nanoparticles and optimize the charge‐discharge performance and cyclability of the obtained anodes.

Supercritical CO_2_ (SCCO_2_) synthesis could be an attractive strategy for producing SiOC. SCCO_2_ fluid is characterized by extremely low viscosity, gas‐like diffusivity, and exceptional mass transfer properties.^[^ ^29^ ^]^ Its near‐zero surface tension allows SCCO_2_ to infiltrate the framework and separate the reactant Si particles. Then, SCCO_2_ could interact with the particles to create a coating layer. Temperature and pressure can be tuned to alter the physicochemical properties of SCCO_2_, such as density, viscosity, and the dielectric constant.^[^ ^30^, ^31^ ^]^ Thus, the characteristics of coating layers can be controlled. Moreover, SCCO_2_ is stable, nonflammable, nontoxic, and inexpensive, making the synthesis process eco‐friendly and scalable.^[^ ^32^ ^]^ SCCO_2_ fluid has been used for extraction since 1950. Large‐scale extraction apparatuses are used in various process plants worldwide.^[^ ^33^, ^34^ ^]^ SCCO_2_ is also used for drying and cleaning in microelectromechanical systems^[^ ^35^ ^]^ and for dye modification treatment in the textile industry.^[^ ^36^ ^]^ For battery material and electrochemistry applications, SCCO_2_ has been used to exfoliate layered materials,^[^ ^37^, ^38^ ^]^ disperse nanostructure materials,^[^ ^39^ ^]^ carry precursors for various material syntheses,^[^ ^40^, ^41^, ^42^ ^]^ and as a precursor in producing porous carbonate nanocomposites.^[^ ^43^ ^]^ Recently, SCCO_2_ fluid has been employed to carry carbohydrate precursors for carbon coating on Si and Li_4_Ti_5_O_12_ particles.^[^ ^44^, ^45^ ^]^ To the best of our knowledge, SCCO_2_ has not been previously used as a precursor to synthesize an SiOC layer on Si particles. We believe this technique is worth developing.

**Scheme 1:**
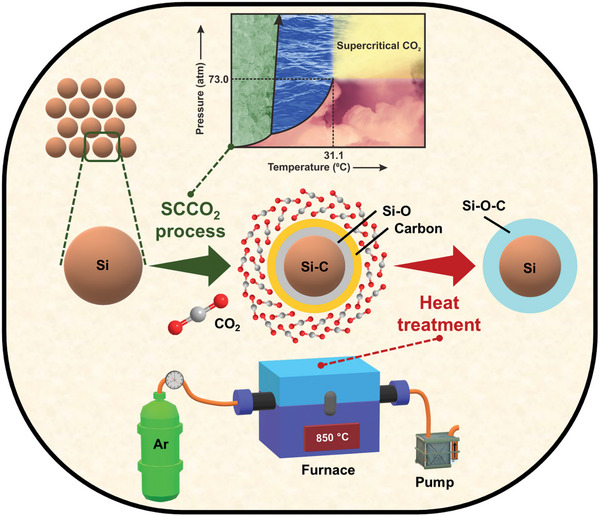
Schematic illustration of combined SCCO_2_ and annealing process for fabrication of core‐shell Si@SiOC composite particles.

In the present work, core‐shell Si@SiOC nanoparticles are synthesized via a facile combined SCCO_2_ and annealing process, as shown in **Scheme** **1**. The reaction time between SCCO_2_ and Si particles is adjusted to optimize the SiOC properties. Samples subjected to only SCCO_2_, or annealing treatment are also prepared for comparison. The microstructure, crystallinity, chemical composition, SEI chemistry, charge–discharge performance, impedance characteristics, and Li^+^ transport properties of various electrodes are systematically studied. In addition, operando X‐ray diffraction (XRD) is used to examine the effects of the SiOC coating on electrode lithiation/delithiation behavior. In addition to a half‐cell investigation, full cells using LiNi_0.8_Co_0.1_Mn_0.1_O_2_ cathodes are constructed and their energy density and cycle life are evaluated. It is shown that the SiOC layer created via the SCCO_2_/annealing process significantly improves the rate capability, Li^+^ transport kinetics, phase transition reversibility, and cyclability of the electrode, which is crucial for next‐generation LIBs.

## 2 Results and Discussion

**Figure 1:**
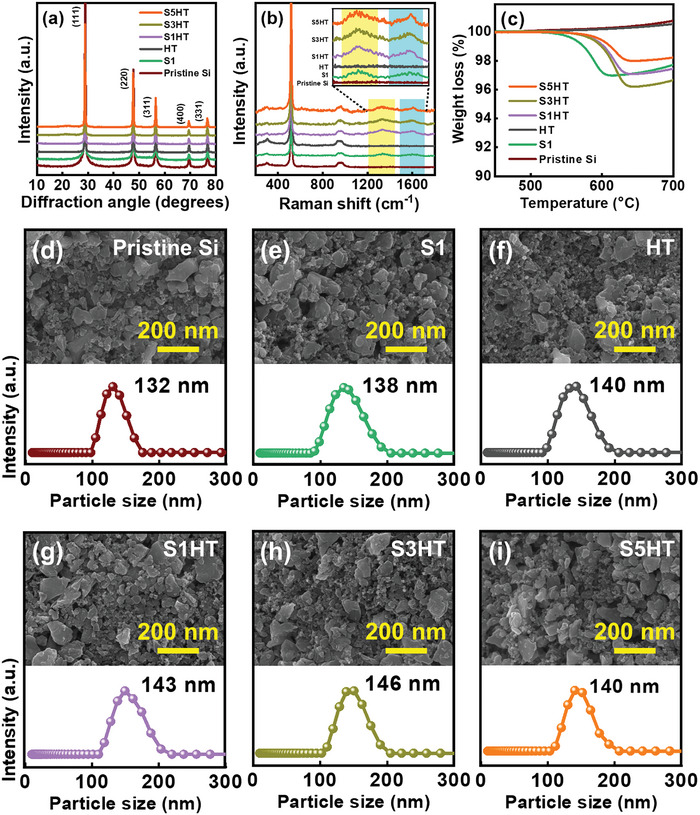
a) XRD, b) Raman, c)TGA, and d–i) SEM/DLS data for pristine Si, S1, HT, S1HT, S3HT, and S5HT samples.

The crystallinity of the pristine Si, S1, HT, S1HT, S3HT, and S5HT samples (S1, S3, and S5 stand for reaction with SCCO_2_ for 1, 3, and 5 h, respectively; HT stands for 850 °C treatment for 5 h. See Section 4.1 for details.) was examined using XRD; the obtained diffraction patterns are shown in **Figure** **1**a. The peaks at 28.4°, 47.4°, 56.1°, 69.2°, and 76.3° belong to the (111), (220), (311), (400), and (331) plane diffraction, respectively, of cubic‐structure Si (JCPDS 27–1402). No signals related to other compounds were detected, indicating that there was no long‐range‐ordering crystalline phase formation after the various treatments. Figure 1b shows the obtained Raman spectra of the samples. The peaks at ≈300, 520, and 960 cm^−1^ are associated with the vibration bands of polycrystalline Si.^[^ ^46^ ^]^ It is noted that the S1, S1HT, S3HT, and S5HT samples exhibit a *D*‐band signal at ≈1340 cm^−1^ and a *G*‐band signal at ≈1605 cm^−1^, which are associated with the existence of a carbon phase. The former signal is related to defective carbon bonding and the latter results from the Raman‐allowed in‐plane vibration of sp^2^ carbon bonding.^[^ ^47^ ^]^ The *D*‐to‐*G*‐band intensity ratio (*I*_D_/*I*_G_) of the S1 sample is ≈1.1. The ratios of the S1HT, S3HT, and S5HT decrease to ≈1.02, reflecting that the ordering of carbon atoms was improved after annealing. Figure 1c shows the thermogravimetric analysis (TGA) data, which quantitatively determine the carbon content of the samples. The observed weight loss at ≈550−650 °C is related to the burnout of the carbon phase.^[^ ^48^ ^]^ The results indicate that the carbon content levels of S1, S1HT, S3HT, and S5HT are ≈3 wt.%, 3 wt.%, 4 wt.%, and 2 wt.%, respectively.

Figure 1d−i shows the morphology and particle size distribution of various samples examined using scanning electron microscopy (SEM) and dynamic light scattering (DLS), respectively. Due to the mechanical milling, irregular particle shapes were observed. However, there was no significant difference in the morphology between the samples. As shown, the *D*_50_ values for the pristine Si, S1, HT, S1HT, S3HT, and S5HT powders are 132, 138, 140, 143, 146, and 140 nm, respectively. After the various treatments, the diameters of the Si particles generally increased, especially after the combined SCCO_2_ and heat treatment (S+HT). This suggests that some reaction products formed on the particle surface. The tap densities of these powders were found to be ≈0.28, 0.45, 0.66, 0.73, 0.78, and 0.71 g cm^−3^, respectively. Although the particle sizes are similar, the S+HT process effectively reduced the electrostatic repulsion between the Si particles, leading to an increased tap density. It is noted that the density of S3HT is higher than those of most silicon‐based nanoparticles reported in the literature.^[^ ^49^, ^50^, ^51^, ^52^, ^53^ ^]^ A dense material is essential for making a compact electrode, which is crucial for electrode volumetric performance.^[^ ^54^ ^]^

**Figure 2:**
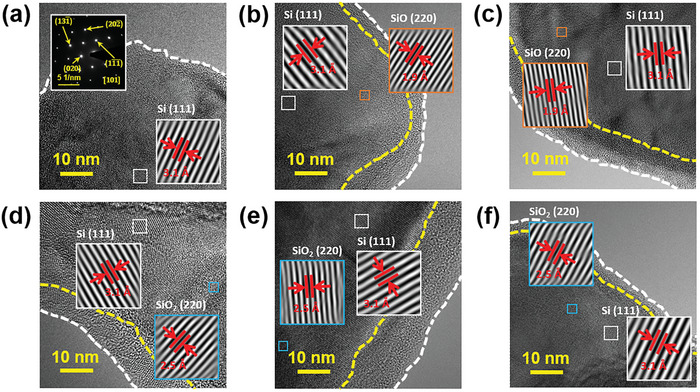
High‐resolution TEM analyses for a) pristine Si, b) S1, c) HT, d) S1HT, e) S3HT, and f) S5HT samples.

**Figure** **2** shows the high‐resolution transmission electron microscopy (TEM) images of all tested samples. The high crystallinity of the pristine Si is confirmed by the lattice image and electron diffraction pattern shown in the insets of Figure 2a. The other samples are covered by amorphous surface layers after the various treatments. As shown in Figure 2b,c, an SiO phase (JCPDS 30–1127), in addition to the Si phase, is dispersed in the S1 and HT samples, reflecting partial oxidation of Si particles during either the SCCO_2_ or annealing process. Figure 2d–f shows the TEM micrographs of the S1HT, S3HT, and S5HT samples, respectively. The lattice images shown in the insets confirm the existence of crystalline SiO_2_ (JCPDS 89–3435). This indicates that further oxidation occurred with the combined S+HT process. The SiO_2_ nanocrystals are thought to be dispersed in the amorphous SiOC phase.

**Figure 3:**
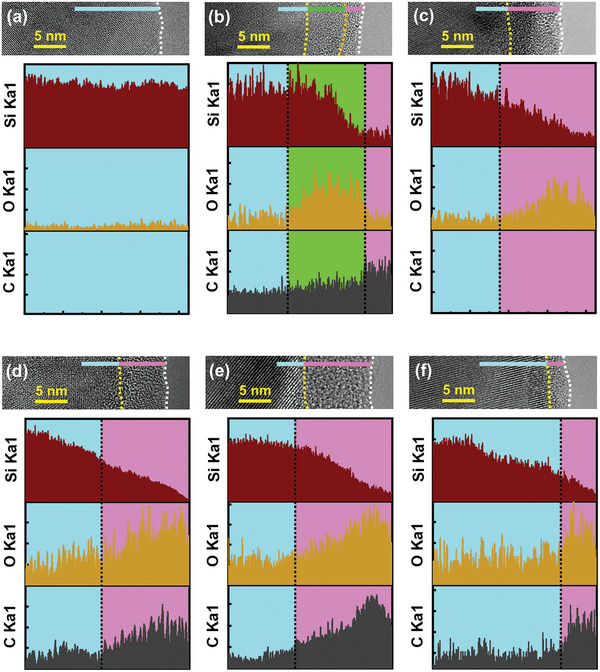
EDS line‐scan data for a) pristine Si, b) S1, c) HT, d) S1HT, e) S3HT, and f) S5HT samples.

**Figure** **3** shows the energy‐dispersive X‐ray spectroscopy (EDS) line‐scan data for various samples. Figure 3a reveals that the pristine Si has no surface coating layer. The observed oxygen signals across the particle are attributed to the surface native oxide. As shown in Figure 3b, after SCCO_2_ treatment, the Si oxidized. A deposited carbon layer was found on the top of the S1 particle. Of note, the carbon concentration in the Si core is considerable. This implies that the carbon from high‐pressure SCCO_2_ dissolved in the Si lattice and formed a metastable Si_1−_ *_y_* C*_y_* solid‐solution phase.^[^ ^55^ ^]^ Figure 3c shows the data for the HT sample, on which a clear surface oxidation layer formed during annealing. The carbon signal intensity for this sample is negligible. This confirms that the detected carbon in Figure 3b was introduced by SCCO_2_. The EDS line‐scan data for S1HT, S3HT, and S5HT, respectively shown in Figure 3d–f, indicate the formation of SiOC layers on the Si cores. After annealing, further oxidation occurred (since the oxygen concentration on the particles had increased compared to that of S1) and the carbon distribution changed. At high temperatures, the dissolved carbon in the metastable Si_1−_ *_y_* C*_y_* is thermodynamically unstable and thus expelled, leading to the formation of surface SiOC layers upon oxidation.

According to the TEM images in Figure 2 and Figure 3, the SiOC layer thickness of S5HT is smaller than that of S3HT. The prolonged SCCO_2_ treatment seems to be detrimental to the development of SiOC. To clarify the cause, commercial SiO_2_ powder was dispersed in SCCO_2_ fluid for various periods of time. Figure S1 (Supporting Information) shows clear weight loss and a particle size reduction of the SiO_2_ upon long exposure to SCCO_2_. The pressure‐induced chemical reaction between SiO_2_ and CO_2_ has been documented in the literature.^[^ ^56^ ^]^ The remarkable affinity between these two substances leads to the formation of silicon carbonate, which could be dissolved or peeled off in SCCO_2_, leading to the observed material loss.

**Figure 4:**
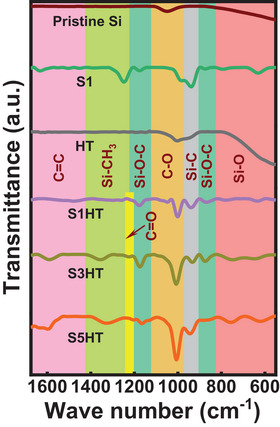
FTIR data for pristine Si, S1, HT, S1HT, S3HT, and S5HT samples.

The functional groups of various samples were evaluated using Fourier‐transform infrared spectroscopy (FTIR); the obtained data are shown in **Figure** **4**. Compared to pristine Si, the S1 sample has elevated peaks of C = C, Si‐CH_3_, and Si‐O, indicating the deposition of carbon and the partial oxidation of Si. The ethanol (used as a co‐solvent with SCCO_2_) may react with Si to form Si‐CH_3_.^[^ ^57^, ^58^ ^]^ The Si‐O‐C peak is weak, whereas the peak corresponding to the Si‐C bond is strong, which supports the presence of the Si_1−_ *_y_* C*_y_* phase (as indicated by the EDS line‐scan data). For HT, the Si‐O is the major species on the particle surface. For the S+HT samples, Si‐O‐C signals clearly appear at 875 and 1175 cm^−1^, confirming the evolution of the surface SiOC layers.^[^ ^59^, ^60^ ^]^ The 1175 cm^−1^ signal can be also attributed to Si‐O‐Si,^[^ ^61^ ^]^ which is associated with the TEM results in Figure 2. The decreased Si‐O‐C intensity of the S5HT sample is in line with the reduced SiOC layer thickness found in the TEM observation. The small signal located at ≈1230 cm^−1^ can be ascribed to the C = O group.^[^ ^62^ ^]^ Moreover, the strong C‐O peak of S5HT is associated with the noticeable affinity of SCCO_2_ toward the Si oxide surface after extended interaction.

**Figure 5:**
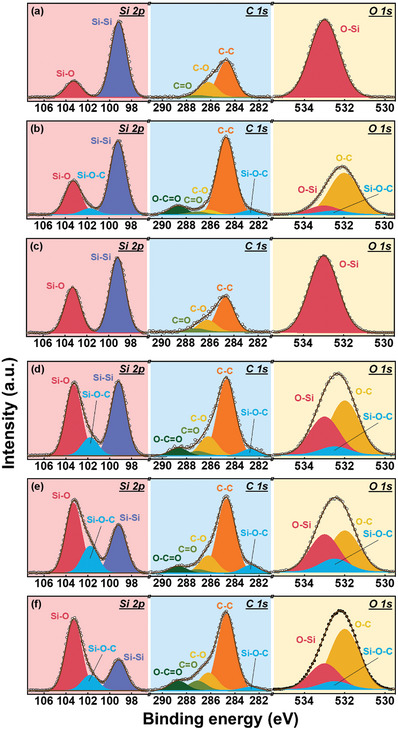
XPS data for a) pristine Si, b) S1, c) HT, d) S1HT, e) S3HT, and f) S5HT samples.

**Figure** **5** shows the X‐ray photoelectron spectroscopy (XPS) data for various samples. As shown, the Si 2p spectra can be deconvoluted into several components. The characteristic peaks at 99.2 and 103.3 eV are associated with Si‐Si and Si‐O species, respectively.^[^ ^63^, ^64^ ^]^ The signal centered at ≈101.8 eV is ascribed to the non‐stoichiometric SiOC.^[^ ^65^, ^66^ ^]^ The S1 sample shows higher Si‐O intensity than that of the pristine Si, indicating oxidation due to the oxidative atmosphere of SCCO_2_. A possible oxidation mechanism is as follows.^[^ ^45^, ^67^ ^]^

(1) $$2\mathrm{SCC}O_{2}⇌O_{2\left(I\right)}+2CO_{\left(g\right)}$$

(2) $$Si_{\left(s\right)}{+}^{x}{/}_{2}O_{2\left(I\right)}⇌\mathrm{Si}O_{x}$$

The O_2(I)_ represents the interstitial oxygen molecule within the oxide. The Si‐O‐C peak intensity of the S1 sample is weak, revealing that the SiOC phase was not well developed before annealing. For the HT sample, the Si‐O peak is strong and there is no Si‐O‐C signal, as expected. After the combined S+HT process, the Si‐O peak grows, and the Si‐O‐C signal becomes pronounced. The S3HT sample has the highest Si‐O‐C intensity among the tested samples, which is consistent with the TEM and FTIR data. As shown in Figure 5, the XPS C 1s spectra are composed of five constituents. In addition to the C‐C peak at 284.7 eV, Si‐O‐C, C‐O, C = O, and O‐C = O signals at 282.7, 286.2, 287.1, and 288.7 eV, respectively, appear.^[^ ^45^, ^68^, ^69^, ^70^ ^]^ Of note, the Si‐O‐C and O‐C = O species are found only for the samples that underwent the SCCO_2_ process. It is also found that the oxygen‐containing functional group concentration on the S1 sample is much lower than those of the other samples. This suggests that there is a carbon‐rich deposit on the S1 sample, rather than the SiOC layers observed on the S+HT samples. The O 1s spectra can be deconvoluted into O‐C, Si‐O‐C, and O‐Si signals at 532.0, 532.5, and 533.0 eV, respectively.^[^ ^71^ ^]^ The data reveal that only the SCCO_2_‐treated samples have the Si‐O‐C compound, whose content is highest in the S3HT sample. This is the first work to produce a SiOC compound using CO_2_; a unique core‐shell Si@SiOC anode material was synthesized using a green and scalable SCCO_2_ process.

The measured electronic conductivity values of the pristine Si, S1, HT, S1HT, S3HT, and S5HT samples are 0.89, 1.01, 0.80, 1.25, 1.48, and 1.19 × 10^−1^ S cm^−1^, respectively. The samples that underwent the S+HT process have relatively high conductivity. The S3HT sample had the best conductivity because it had the thickest SiOC coating. The electronic conductivity values of SiOC reported in the literature are in a range of 10^−13^−10^0^ S cm^−1^, depending on the synthesis conditions and composition.^[^ ^72^, ^73^ ^]^ The SiOC layer formed in this study clearly increases the conductivity of the pristine Si.

Cyclic voltammetry (CV) measurements were performed to examine the electrochemical properties of various electrodes. Figure S2 (Supporting Information) shows the CV curves of the pristine Si, S1, HT, S1HT, S3HT, and S5HT electrodes recorded at 0.1 mV s^−1^. During the first negative scan, cathodic peaks appeared at ≈1.2 and ≈0.1 V for all electrodes. The former peak can be assigned to electrolyte decomposition and SEI formation.^[^ ^74^ ^]^ The latter peak is ascribed to the lithiation of the Si phase and the evolution of various Li‐Si alloys.^[^ ^75^ ^]^ Upon the positive scan, two distinct anodic peaks emerged at ≈0.35 and ≈0.52 V, which corresponded to the phase transition from Li_15_Si_4_ to amorphous Li*_x_* Si and that from amorphous Li*_x_* Si to Si, respectively.^[^ ^76^ ^]^ Because the surface coating layers were quite thin, they did not significantly affect the CV behavior. In the subsequent cycles, the CV shapes varied due to the electro‐activation process.^[^ ^77^ ^]^ Specifically, the cathodic alloying reactions were promoted to less negative potentials, and both the cathodic and anodic current densities increased. It is noted that the S+H electrodes seem to need fewer CV scan numbers to reach the saturation current; the pristine Si, S1, and HT electrodes showed increasing CV current even at the fifth scan. These results reflect the superior electronic and ionic conductivity of the S+H electrodes, which leads to a shorter activation course.

**Figure 6:**
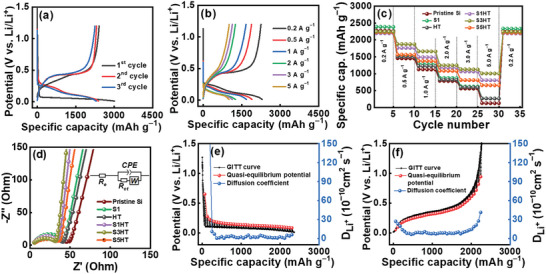
a,b) Charge–discharge curves of S3HT electrode. c) Comparative rate performance and d) EIS spectra of various electrodes. Quasi‐equilibrium potential and *D*_Li_^+^ values of S3HT electrode measured using GITT during e) lithiation and f) delithiation.

**Figure** **6**a and S3 (Supporting Information) show the initial three charge‐discharge curves of various electrodes measured at a current rate of 0.2 A g^−1^. The first‐cycle CE values for the pristine Si, S1, HT, S1HT, S3HT, and S5HT electrodes are 70%, 70%, 68%, 74%, 80%, and 71%, respectively. The S3HT sample has the highest initial CE, which can be attributed to it having the highest electronic conductivity among the samples. Moreover, the surface SiOC layer could maintain particle integrity (minimizing mechanical breakdown). Both factors promote reaction reversibility, enhancing CE. The initial CE for HT is relatively low, which is associated with the irreversible conversion reaction of SiO*_x_* upon first lithiation.^[^ ^78^ ^]^ With the aid of the S+HT‐derived SiOC, the first‐cycle CE of S3HT is among the highest values reported in the literature, as shown in Table S1 (Supporting Information). The carbon incorporation not only reduced the oxygen portion (and thus the irreversible conversion reaction) in the SiOC layer but also created electron‐conducting pathways. The initial CE was thus improved. A high initial CE is crucial for Si‐based anodes and determines their practical applicability.^[^ ^79^ ^]^ Figure 6b and Figure S4 (Supporting Information) show the charge‐discharge profiles of the electrodes measured at various current rates after two conditioning cycles. The reversible capacities obtained at 0.2 A g^−1^ are 2280, 2295, 2259, 2201, 2250, and 2230 mAh g^−1^ for the pristine Si, S1, HT, S1HT, S3HT, and S5HT electrodes, respectively. With increasing current rate, the specific capacities decreased, as shown in Figure 6c and **Table** **1**. The capacity values of these electrodes reduced to 136, 275, 272, 814, 1013, and 668 mAh g^−1^, respectively, at a specific current of 5 A g^−1^, corresponding to 6%, 12%, 12%, 37%, 45%, and 30% of the capacities measured at 0.2 A g^−1^. SCCO_2_ fluid disperses the Si particles well, forming an effective SiOC conducting network in between. Moreover, the thin and conformal SiOC coating makes the particles closely connected (as indicated by the tap density data), which benefits electron and Li^+^ transport. As a result, superior high‐rate performance of the S+H electrodes was achieved. However, prolonged SCCO_2_ reaction time reduced the SiOC thickness and electronic conductivity, decreasing the electrode rate capability.

**Table 1:** Reversible specific capacities of pristine Si, S1, HT, S1HT, S3HT, and S5HT electrodes measured at various current rates.

Current rate (A g^−1^)	Pristine Si (mAh g^−1^)	S1 (mAh g^−1^)	HT (mAh g^−1^)	S1HT (mAh g^−1^)	S3HT (mAh g^−1^)	S5HT (mAh g^−1^)
0.2	2280	2295	2259	2201	2250	2230
0.5	1459	1539	1512	1769	1880	1572
1	1136	1301	1234	1497	1666	1382
2	792	882	831	1184	1254	1091
3	566	630	606	1070	1134	817
5	136	275	272	814	1013	668
High rate retention^*^	6%	12%	12%	37%	45%	30%

* a comparison between reversible capacities at 5 and 0.2 A g^−1^.

Figure 6d shows the electrochemical impedance spectroscopy (EIS) data for various electrodes acquired after two conditioning cycles. The Nyquist spectra consist of a semicircle at high frequency followed by a sloping line at low frequency, which can be characterized by the equivalent circuit shown in the figure inset, where *R*_e_, *R*_ct_, *CPE*, and *W* represent the electrolyte resistance, charge transfer resistance, interfacial constant‐phase element, and Warburg impedance associated with Li^+^ diffusion within the electrode, respectively.^[^ ^41^ ^]^ As shown in **Table** **2**, the *R*_ct_ values are 40, 36, 35, 28, 26, and 30 Ω for the pristine Si, S1, HT, S1HT, S3HT, and S5HT electrodes, respectively. S3HT having the lowest *R*_ct_ can be attributed to its superior SiOC layer quantity and optimal electronic conductivity. The galvanostatic intermittent titration technique (GITT) was used to evaluate the Li^+^ transport properties of the electrodes. Figure 6e,f show the obtained lithiation and delithiation data for the S3HT electrode; the data for the other electrodes are shown in Figure S5 (Supporting Information). The calculated average *D*_Li_^+^ values are summarized in Table 2. The S3HT electrode has the highest *D*_Li_^+^ values (i.e., 7.6 and 8.1 × 10^−10^ cm^2^ s^−1^ for lithiation and delithiation, respectively), followed by the S1HT, S5HT, HT, S1, and then pristine Si electrodes. The *R*_ct_ and *D*_Li_^+^ data explain the rate capability variation between the electrodes.

**Table 2:** *R*_ct_ and *D*_Li_^+^ values of pristine Si, S1, HT, S1HT, S3HT, and S5HT electrodes.

Sample	*R*_ct_ after conditioning cycles (Ω)	*R*_ct_ after 300 cycles (Ω)	Lithiation/Delithiation *D*_Li_^+^ (× 10^−10^ cm^2^ s^−1^)
Pristine Si	40	77	1.4/1.9
S1	36	69	2.8/3.0
HT	35	65	2.6/2.9
S1HT	28	47	6.8/7.2
S3HT	26	43	7.6/8.1
S5HT	30	53	5.7/6.4

**Figure 7:**
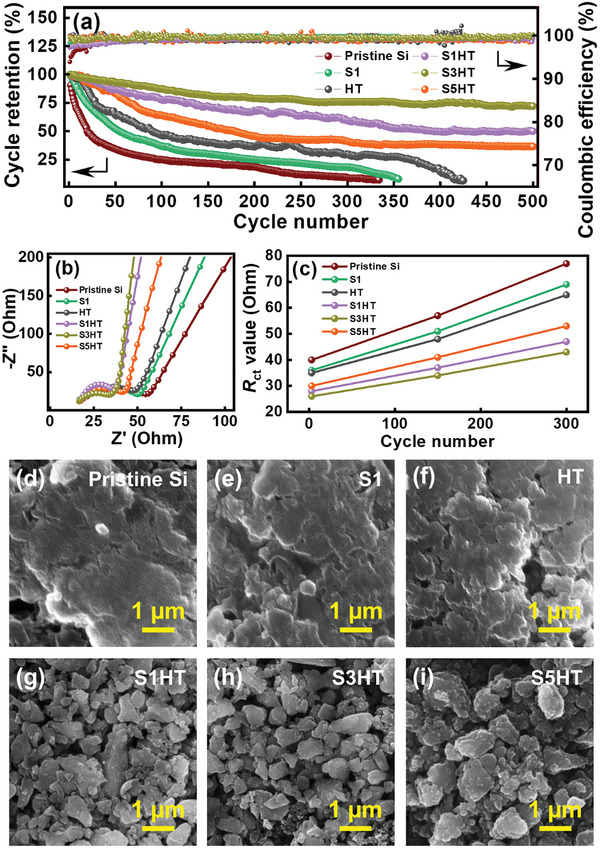
a) Cycling stability data for various electrodes measured at 0.5 A g^−1^. b) EIS spectra of various electrodes acquired after 300 charge–discharge cycles. c) Variation of *R*_ct_ values of various electrodes with respect to charge‐discharge cycle number. d–i) Postmortem SEM images of various electrodes taken after 300 charge–discharge cycles.

**Figure** **7**a shows the cycling stability data for various electrodes measured at 0.5 A g^−1^. The pristine Si and S1 cells showed almost no capacity after 300 cycles and the HT cell failed after 400 cycles. In contrast, after 500 charge‐discharge cycles, the S1HT, S3HT, and S5HT electrodes retained 50%, 72%, and 36% of their initial capacities, respectively. As compared in Table S1 (Supporting Information), the cycling stability of the S3HT electrode is among the best reported for Si/SiOC composite anodes. Even under harsh conditions (a capacity of 1880 mAh g^−1^ and a rate of 0.5 A g^−1^), where substantial and fast electrode volume change occurred, satisfactory cyclability was achieved for the S3HT electrode, which showed a steady CE of >99.8% up to 500 cycles. Note that we did not optimize the binder and electrolyte recipes and did not use any sophisticated electrode architectures to maximize cycle life. Therefore, further improvement in electrode cycling stability using additional strategies is expected. Figure 7b shows the EIS spectra of the electrodes after 300 charge‐discharge cycles. The Nyquist circles clearly evolve upon cycling. As shown in Table 2, the *R*_ct_ values increase to 77, 69, 65, 47, 43, and 53 Ω for the pristine Si, S1, HT, S1HT, S3HT, and S5HT electrodes, respectively, after 300 cycles. The relatively large increase of *R*_ct_ for the pristine Si, S1, and HT electrodes (shown in Figure 7c) can be rationalized based on the postmortem SEM images shown in Figure 7d–f. The morphologies of these electrodes are significantly distorted compared to those of the original electrodes (see Figure 1). The Si particles greatly expanded and agglomerated. Moreover, their surfaces were covered by thick SEI layers. Therefore, the charge transfer reactions were hindered. In contrast, the structures of the S+HT electrodes were highly preserved after cycling (Figure 7g–i), explaining the superior *R*_ct_ and capacity stability.

The corrosion resistance of the pristine Si and S3HT samples to HF, a corrosive species that is usually present in a battery, was evaluated. Both kinds of powder were immersed in 25 mM HF solution at 25 °C for 1 h.^[^ ^70^ ^]^ The weight and particle size variations were measured; the data are shown in Figure S6 (Supporting Information). It was confirmed that the dissolution rate of S3HT is significantly lower than that of pristine Si due to the existence of the SiOC protection layer.

**Figure 8:**
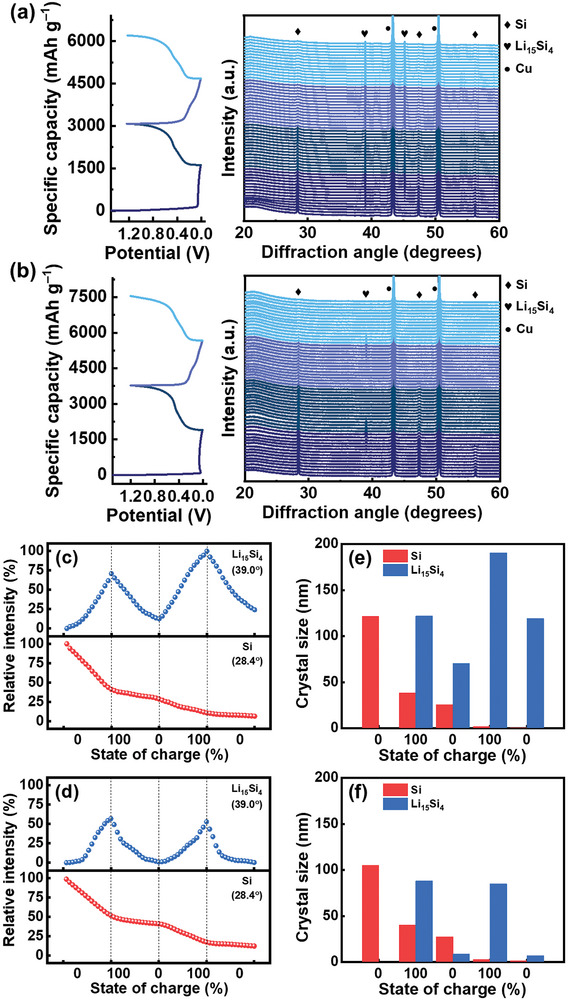
Operando XRD data for a) pristine Si and b) S3HT electrodes during initial two charge–discharge cycles. Relative Si and Li_15_Si_4_ peak intensity versus state of charge for c) pristine Si and (d) S3HT electrodes. Crystal size variations of Si and Li_15_Si_4_ phases for e) pristine Si and f) S3HT electrodes.

**Figure** **8**a,b show the operando XRD data for the pristine Si and S3HT electrodes, respectively, during the initial two charge‐discharge cycles. In addition to the Cu substrate peaks, signals associated with Si and Li_15_Si_4_ phases were observed. In general, the intensity of the crystalline Si phase continuously diminished due to the amorphization during lithiation.^[^ ^80^ ^]^ The formation and elimination of the Li_15_Si_4_ phase were observed upon lithiation and delithiation processes, respectively. As shown in Figure 8c, for the pristine Si electrode, after delithiation (or discharge), the Li_15_Si_4_ phase was preserved. This indicates that the reversibility was not complete. Moreover, the Li_15_Si_4_ intensity in the second cycle is considerably higher than that in the first cycle, indicating crystal growth and agglomeration. As shown in Figure 8d, much better reversibility of Li_15_Si_4_ formation and dissolution upon cycling was found for the S3HT electrode. Basically, the Li_15_Si_4_ formed during lithiation was completely eliminated during delithiation. Of note, the Li_15_Si_4_ crystalline orientation is different in the two electrodes (Figure 8a,b). For the pristine Si electrode, both (332) and (431) peaks of Li_15_Si_4_ appear, whereas the S3HT electrode shows only a (332) diffraction peak. This can be attributed to the existence of the surface SiOC layer, which alters the Li^+^ flux and Li_15_Si_4_ nucleation characteristics. It was reported^[^ ^81^ ^]^ that a part of SiO_4_ units of SiOC can be reversibly converted to Li_2_SiO_3_ during charging/discharging, while the others are irreversibly transformed to Li_4_SiO_4_. Besides, the SiOC_3_ units are totally irreversible; they disappear in the first lithiation process and lead to the formation of SiC_4_ units. These new species could affect the crystalline orientation of the Li_15_Si_4_ phase during the charge‐discharge process. In addition, the residual carbon dissolved in the Si phase (caused by SCCO_2_) may alter the physicochemical properties of Si, which changes Li_15_Si_4_ phase formation behavior. Further mechanistic studies are needed. The effect of the Li_15_Si_4_ crystalline orientation on the electrochemical properties also deserves future investigation.

The crystal size (*L*) of the Si and Li_15_Si_4_ phases at various states of charge can be estimated using Scherrer's formula:^[^ ^82^ ^]^

(3) $$L\;=\frac{K\times \lambda }{B\;\cos\theta \;}\;$$

where *K* is the Scherrer constant (0.94 is adopted in this study), *λ* is the X‐ray wavelength, and *B* is the full width at half maximum of the XRD peak at a diffraction angle of 2*θ*. The calculation results for the pristine Si and S3HT electrodes are shown in Figure 8e,f, respectively. While the crystal size of the Si phase monotonously reduces, fluctuation of the *L* value for Li_15_Si_4_ is noted. As shown, with the incorporation of the SiOC layer, the crystal size of Li_15_Si_4_ becomes markedly smaller, and its variation upon lithiation and delithiation becomes more reversible. This explains the enhanced cyclability of the S3HT electrode compared to that of the pristine Si electrode.

**Figure 9:**
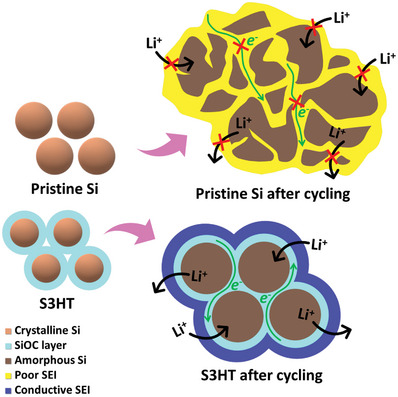
Schematic illustration of structure evolution of pristine Si and S3HT electrodes after charge‐discharge cycling.

**Figure** **9** shows the structure evolutions of the pristine Si and S3HT electrodes upon cycling. The former electrode has low electronic conductivity and loose interparticle connectivity. The large volume variation of the Si particles during lithiation/delithiation causes serious mechanical degradation. The repeated breakdown and reformation of the SEI lead to an increase in its thickness, which could isolate the Si particles and hinder Li^+^ transport, resulting in rapid electrode performance deterioration. In contrast, for S3HT, with the aid of SCCO_2_, the Si particles are well dispersed and uniformly coated by a SiOC layer. The conformal and continuous SiOC not only forms an electronic conducting network within the electrode but also ensures a close connection of the Si particles, which enhances Li^+^ conduction. Moreover, this resilient SiOC layer can buffer the Si volume change and wrap the particles to prevent their pulverization.^[^ ^21^ ^]^ Consequently, the interfacial SEI can be stabilized and good cyclability can be achieved. Figure S7 (Supporting Information) compares the cross‐section SEM images of the pristine Si and S3HT electrodes before and after 50 charge‐discharge cycles. Much less irreversible volume expansion and better electrode integrity are found for the S3HT electrode. Figure S8 (Supporting Information) compares the XPS data for the S3HT electrode after two conditioning cycles and those after 300 charge–discharge cycles. As shown, the SEI consists of Li*_x_* SiO*_y_*, LiF, Li_2_CO_3_, Li‐O, C‐F, and Li*_x_* PO*_y_* F*_z_* species. It is found that the two sets (acquired after the 2nd and 300th cycles) of XPS data are close to each other. This indicates that the SEI layer on the S3HT electrode is robust and stable against long cycling.

**Figure 10:**
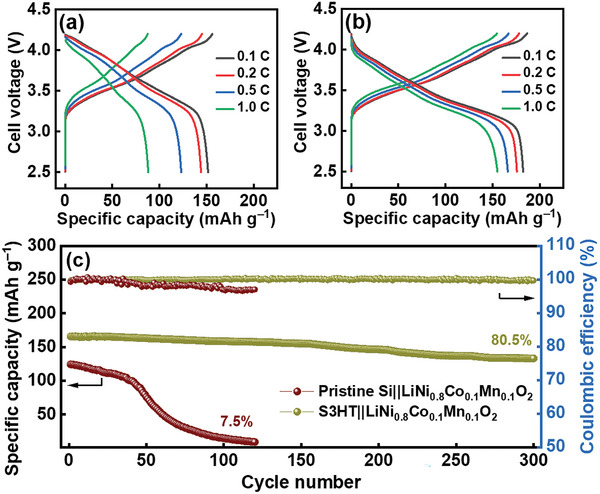
Charge‐discharge profiles of a) pristine Si||LiNi_0.8_Co_0.1_Mn_0.1_O_2_ and b) S3HT||LiNi_0.8_Co_0.1_Mn_0.1_O_2_ full cells measured at various current rates after two conditioning cycles. c) Cycling stability of the two cells measured at 0.5 C.

Pristine Si||LiNi_0.8_Co_0.1_Mn_0.1_O_2_ and S3HT||LiNi_0.8_Co_0.1_Mn_0.1_O_2_ full cells were constructed with an anode‐to‐cathode capacity ratio of 1.15. The negative electrode was prelithiated to 10% capacity in a half cell prior to the full‐cell assembly. **Figure** **10**a,b show the charge‐discharge profiles measured at various current rates after two conditioning cycles performed at 0.1 C (1 C = 200 mA g^−1^ for LiNi_0.8_Co_0.1_Mn_0.1_O_2_). As shown in Table S2 (Supporting Information), the reversible specific capacities (based on both the anode and cathode masses) of the latter cell are 182, 176, 166, and 165 mAh g^–1^ at 0.1, 0.2, 0.5, and 1 C, respectively, which are clearly higher than those of the former cell. The gravimetric energy density of the S3HT||LiNi_0.8_Co_0.1_Mn_0.1_O_2_ cell calculated based on the discharge profile at 0.1 C is ≈600 Wh kg^−1^, where the kg includes the weights of the cathode and anode active materials, but not the electrolyte, current collectors, binders, conductive agents, or the separator used. The superior energy density indicates the merit of the proposed S3HT anode. Figure 10c shows the cycling stability of the two cells measured at 0.5 C. The Si||LiNi_0.8_Co_0.1_Mn_0.1_O_2_ cell has little capacity after ≈120 cycles, whereas the S3HT||LiNi_0.8_Co_0.1_Mn_0.1_O_2_ cell retains more than 80% capacity after 300 charge‐discharge cycles. The Si particles with an SCCO_2_‐derived SiOC coating have great electrochemical stability for high‐energy‐density LIB applications.

## 3 Conclusions

A unique combined SCCO_2_ and annealing process was developed to synthesize core‐shell Si@SiOC particles. During the SCCO_2_ process, in addition to SiO formation, the carbon dissolves in the Si lattice and becomes deposited on the Si particles. Upon annealing, further oxidation occurs and the dissolved carbon in the metastable Si_1−_ *_y_* C*_y_* is expelled, leading to the development of an amorphous SiOC shell at high temperature. An SCCO_2_ reaction time of 3 h was found to be appropriate. Prolonging the reaction time reduces the SiOC layer thickness, probably due to the formation of silicon carbonate, which dissolves or peels off in SCCO_2_. The conducting SiOC layer brings the Si@SiOC particles closer together, benefiting electron and Li^+^ transport. In addition, the buffering SiOC layer can accommodate the Si volume change during lithiation/delithiation. Of note, the protective SiOC layer provides high corrosion resistance to HF. As a consequence, the Si@SiOC electrode shows specific capacities of 2250 and 1013 mAh g^−1^ at 0.2 and 5 A g^−1^, respectively. After 500 charge–discharge cycles at 0.5 A g^−1^, the capacity retention was 72% (with a CE of above 99.8%). Operando XRD data indicate that the crystal size and orientation of the lithiated Li_15_Si_4_ phase were altered by the presence of SiOC, which promoted phase transition reversibility during cycling. Much less irreversible volume expansion and better electrode integrity after cycling were confirmed by the postmortem cross‐section SEM analysis of the S3HT electrode compared to those of the pristine Si electrode. Moreover, the XPS data for the S3HT electrode revealed that the SEI is stable after long cycling. The S3HT||LiNi_0.8_Co_0.1_Mn_0.1_O_2_ full cell showed a promising energy density of ≈600 Wh kg^−1^ (based on anode and cathode active materials) with satisfactory cyclability. The proposed SCCO_2_ synthesis process is eco‐friendly, cost‐effective, and scalable. The obtained Si@SiOC anode material has great potential for high‐energy‐density and high‐reliability LIB applications.

## 4 Experimental Section

### Creation of SiOC Shell on Si Particles

Micrometer‐size Si powder (*D*_50_: 1.8 µm; purity > 99.9%) was provided by Super Energy Material Inc., Taiwan. Planetary ball milling was conducted for 24 h to reduce the *D*_50_ value to ≈130 nm. The Si powder was then dispersed in an anhydrous ethanol solution and transferred into an SCCO_2_ reactor. Ethanol, which is miscible with SCCO_2_, was used as a co‐solvent to facilitate the handling and collection of the powdery samples. The chamber was pressurized with CO_2_ up to 8 MPa at ≈55 °C, at which point the CO_2_ reached a supercritical state. The system was stirred vigorously for 1, 3, and 5 h, respectively, before depressurization. The resulting powder was dried at 65 °C overnight. Afterward, the powder was heated at 850 °C under an Ar flow for 5 h. The synthesis procedures are shown in Scheme 1. The obtained samples are denoted as S1HT, S3HT, and S5HT, respectively. For comparison, samples subjected to only 1‐h SCCO_2_ treatment (without annealing) and only 850 °C heat treatment (without the SCCO_2_ process) were fabricated; they are denoted as S1 and HT, respectively.

### Cell Assembly

The anode active material, conducting Super P, and sodium polyacrylate binder were mixed in an 80:10:10 weight ratio in deionized water. This slurry was cast onto Cu foil using a doctor blade and vacuum‐dried at 100 °C for 8 h. The obtained electrodes were punched to match the required dimensions of a CR2032 coin cell. The active material mass loading was ≈2 mg cm^−2^. Li foil and a glass fiber membrane were used as the counter electrode and separator, respectively. For full‐cell assembly, the S3HT negative electrode was paired with a LiNi_0.8_Mn_0.1_Co_0.1_O_2_ (NMC‐811) positive electrode with a capacity ratio of 1.15:1. The anode was prelithiated to 10% capacity in a half cell prior to the full‐cell assembly. An electrolyte composed of 1 M LiPF_6_ salt, ethylene carbonate/diethyl carbonate mixed solvent (1:1 by volume), and 10 wt.% fluoroethylene carbonate was adopted. The coin cells were assembled in an Ar‐filled glove box (Vigor Tech. Co. Ltd.), where the moisture and oxygen content levels were maintained at ≈0.1 ppm.

### Material and Electrochemical Characterizations

The crystallinity of the samples was characterized using XRD (Bruker D2 Phaser). The Raman spectra were collected using a spectrometer (LabRAM HR 800) with an excitation laser wavelength of 633 nm. TGA (TA Instruments Q500) was conducted under air with a heating rate of 5 °C min^−1^. The morphology, microstructure, and chemical composition of the samples were examined using scanning electron microscopy (SEM; JEOL JSM7800F Prime), TEM (JEOL F200), and their auxiliary EDS. The functional groups on the samples were analyzed using FTIR (PerkinElmer Spectrum 100). XPS (Thermo Fisher Scientific ESCALAB Xi^+^) was employed to analyze the surface chemistry. Al K_α_ radiation (1486.6 eV) was adopted as the X‐ray excitation source. The C 1s signal at 284.7 eV was used for binding energy calibration. The data fitting was done using the software XPSPEAK 4.1. The particle size of the samples was estimated using DLS (Otsuka ELSZ‐2000), in which ethanol was used as a dispersant. CV (BioLogic BCS‐810) was performed in a range of 0.01–2.0 V (versus Li/Li^+^) with a potential scan rate of 0.1 mV s^−1^. EIS was conducted within a frequency range of 10^6^–10^−2 ^Hz using a potential perturbation amplitude of 10 mV. The charge‐discharge properties, such as capacity, rate capability, and cycling stability, of various cells were evaluated using a battery tester (Arbin BT‐2043) at 25 °C. GITT was used to assess the apparent Li^+^ diffusion coefficient (*D*_Li_^+^) of various electrodes. For operando XRD analyses, the cells were subjected to synchrotron X‐ray examination during charging/discharging at a rate of 0.5 A g^−1^. These analyses were performed at Beamline TPS‐19A of the National Synchrotron Radiation Research Center, Taiwan.

### Statistical Analysis

The CV, EIS, GITT, and charge–discharge measurements of various electrodes were repeated at least three times to ensure validity. The data deviation was typically within ≈3% and the reported values are the medians. All the XPS spectra were calibrated with the binding energy of C 1s peak at 284.7 eV. The data fitting was done using XPSPEAK 4.1 software. For XRD data, the background subtraction and phase identification were conducted using the EVA and TOPAS programs provided in the Bruker software package. The Origin software was used for data analysis and processing.

## Conflict of Interest

The authors declare no conflict of interest.

## Supporting information

Supporting Information

## Data Availability

The data that support the findings of this study are available from the corresponding author upon reasonable request.
