# Supplementary material for: Core‐Shell Si@SiOC Particles Synthesized Using Supercritical Carbon Dioxide Fluid for Superior Li‐Ion Storage Performance
Source: Adv Sci (Weinh). 2024 Jun 17;11(31):2401350. doi: 10.1002/advs.202401350 (PMC11336897; doi:10.1002/advs.202401350)
Supplement: Supplementary file 1 — Supporting Information [file ADVS-11-2401350-s001.docx]

Copyright WILEY-VCH Verlag GmbH & Co. KGaA, 69469 Weinheim, Germany, 2024.

Supporting Information

**Core-Shell Si@SiOC Particles Synthesized Using Supercritical Carbon Dioxide Fluid for Superior Li-Ion Storage Performance**

*Rahmandhika Firdauzha Hary Hernandha, Bharath Umesh,* *Jagabandhu Patra, Chun-Yen Chen, Ju Li, Jeng-Kuei Chang**

**Table S1.** Performance comparison of S3HT electrode with various Si/SiOC composite anodes reported in the literature.

| **Materials** | **Synthesis method** | **Electrolyte** | **Initial delithiation capacity**  **(mAh g^–1^)/ CE (%)** | **Capacity**  **(mAh g^–1^)/**  **cycle number/**  **retention (%)** | **Ref.** |
| --- | --- | --- | --- | --- | --- |
| Si/Si–O–C composite | Mixing Si NPs with triethoxysilane and diethoxymethylsilane. Add diethyl benzene and phenylsubstituted polysiloxane (1:1 weight ratio). Hydrolyze with distilled water (H2O/OR = 1; pH = 4), add 1 ppm chloroplatinic acid for gelation and cross-linking. Pyrolyze at 1100 °C for 1 hour in H_2_ atmosphere. | 1 M LiPF_6_ in EC/DEC (1:1 vol%) | 1371.6/78 | 990/30/73 (@0.05 A g^–1^) | [1] |
| SiOC/nSi_amorphous_ | Mixing nano-amorphous (~36 nm) and nano-crystalline (30-50 nm) silicon particles, separately with polyorganosiloxane in acetone and heat-treated at 1100 °C. | 1 M LiPF_6_ in EC/DMC (1:1 vol%) | ~600/~63 | ~530/100/~88  (@0.074 A g^–1^) | [2] |
| SiOC/nSi_crystalline_ |  |  | ~800/~75 | ~130/100/<17  (@0.074 A g^–1^) |  |
| Si@SiOC | Aerosol-assisted chemical vapor deposition mechanism in phenyltriethoxysilane solution at 800 °C and heat-treated at 800 °C for 1 h under Ar atmosphere. | 1 M LiPF_6_ in EC/DEC (1:1 vol%) + 5 wt% FEC | 2093/72 | ~1500/200/92 (@2 A g^–1^) | [3] |
| Si/C/SiOC | Synthesis Si/C: fructose as carbon precursor dissolved into water-ethanol solution and heat treated at 1100 °C. Then, continuing by mixing the Si/C with preceramic organosilicon polymer in acetone, the 2^nd^ heat treatment is conducted at 1100 °C in Ar. | 1 M LiPF_6_ in EC/DMC (1:1 vol%) | ~2000/~60 | ~2000/100/>100*  (@0.072 A g^–1^)  *the capacity is increasing from 1^st^ to 50^th^ | [4] |
| 3D Si/C/SiOC nanocomposites | Wet chemical mixing used fructose as a C-precursor and heat treated at Ar at 1100 °C. For SiOC coating, nanostructured Si coated with carbon were dispersed in the commercial polysiloxane with acetone, evaporated, and pyrolyzed under Ar at 1100 °C. | 1 M LiPF_6_ in EC/DMC (1:1 vol%) | 533/61 | ~575/50/108*  (@0.072 A g^–1^)  *the capacity is increasing from 1^st^ to 50^th^ | [5] |
| Si_0.6_/SiO composite | Started with synthesize of poly-phenyl-silsesquioxane nanospheres by hydrolysis–condensation process, mixed with SiNPs and direct pyrolysis. | 1 M LiPF_6_ in EC/DEC (3:7 vol%) + 5 wt% FEC | 980/~63 | ~600/500/~80  (@0.5 A g^–1^) | [6] |
| Si/SiOC nanosheets | In situ crosslinking of poly(triethoxysilyl propyl methacrylate) in the solvent via evaporation, following by carbonization at 800 °C for 3 h under air, and magnesiothermic reduction at 300 °C for 20 h under Ar. Finally, washed in HCl overnight. | 1 M LiPF_6_ in EC/DEC (1:1 vol%) + 5 wt% FEC | 1461/58 | 529/500/73 (@5 A g^–1^) | [7] |
| CTAB-Si/SiOC composite | Si NPs mixed with cetrimonium bromide as surfactant in ethanol, the silicone oil was slowly poured into the solution under vigorous stirring in 80 °C for 2 h. Obtained precursor were heat treated at 900 °C and ball milled. | 1.3 M LiPF_6_ in EC/DEC (1:1 vol%) + 5 wt% FEC | 1649/~78.3 | 1312/100/89 (@0.5 A g^–1^) | [8] |
| Si/SiOC thin film | Amorphous silicon and SiOC films were prepared by radio frequency magnetron sputtering and deposited on Cu foils. The commercial Si, C, and SiO_2_ targets with diameters of 50.8 mm and thicknesses of 5 mm were used. The final thickness is approximately 100 nm. | 1 M LiPF_6_ in EC/DEC (1:1 vol%) + 2% VC | ~1900/~80 | ~1400/100/~70 (@0.05 C) | [9] |
| Si/SiO_x_-SiOC/C composite | Mixing hydrocarbon resin, Si powder, and polyacrylic acid followed by a ball milling process and heat treatment at temperatures ranging from 600 °C to 900 °C. | N/A | 1846/~81 | 1402/100/76 (@1 C) | [10] |
| Si/SiOC/C | Thermal polymerization of the composite containing Si NPs, vinyl terminated PDMS, and Bis-GMA, then calcination treatment. | Commercial electrolyte | 452/57.7 | ~377/100/83.4  (@0.5 A g^–1^) | [11] |
| Si/C/SiOC | Started with the dopamine polymerization chemistry at pH 8.5 and heat treatment at 800 °C to achieve C-coating, further, it was introduced in an aqueous dispersion of acetylene black and C_9_H_23_NO_3_Si solution for a night and heat treated at 1000 °C for 10 h at nitrogen ambiance. | 1 M LiPF_6_ in EC/DEC (1:1 vol%) | 957/33.6 | 1017/775/>92  (@0.75 A g^–1^) | [12] |
| Core-Shell Si@SiOC | Single step supercritical carbon dioxide-assisted reaction with miscible absolute ethanol as co-solvent and heat treated under Ar at 850 °C for 5 h. | 1 M LiPF_6_ in EC/DEC (1:1 vol%) + 10 wt% FEC | 2411/80 | ~1690/100/90; ~1500/200/80; ~1410/300/75; ~1355/500/72  (@0.5 A g^–1^) | This work |

**References**

[1] X. Liu, K. Xie, J. Wang, C. Zheng, Y. Pan, *J. Mater. Chem.* **2012**, *22*, 19621–19624.

[2] J. Kaspar, M. Graczyk-Zajac, S. Lauterbach, H. J. Kleebe, R. Riedel, *J. Power Sources* **2014**, *269*, 164−172.

[3] S. Choi, D. S. Jung, J. W. Choi, *Nano Lett.* **2014**, *14*, 7120−7125.

[4] D. Vrankovic, M. Graczyk-Zajac, C. Kalcher, J. Rohrer, M. Becker, C. Stabler, G. Trykowski, K. Albe, R. Riedel, *ACS Nano* **2017**, *11*, 11409−11416.

[5] D. Vrankovic, K. Wissel, M. Graczyk-Zajac, R. Riedel, *Solid State Ion.* **2017**, *302*, 66−71.

[6] Z. Wu, W. Lv, X. Cheng, J. Gao, Z. Qian, D. Tian, J. Li, W. He, C. Yang, *Chem. Eur. J.* **2019**, *25*, 2604 –2609.

[7] J. Huang, K. Leng, Y. Chen, L. Chen, S. Liu, S. Khan, D. Wu, R. Fu, *J. Mater. Chem. A* **2019**, *7*, 22950–22957.

[8] J. Jang, H. Kim, H. Lim, K. J. Kim, H. G. Jung, S. O. Kim, W. Choi, *Chem. Eng. J.* **2020**, *401*, 126091.

[9] W. Wei, K. Xu, N. Liao, W. Xue, *Appl. Mater. Today* **2020**, *20*, 100773.

[10] W. Y. Chung, S. Brahma, S. C. Hou, C. C. Chang, J. L. Huang, *Mater. Chem. Phys.* **2021**, *259*, 124011.

[11] M. Wang, Y. J. Cheng, Y. Xia, *ChemistrySelect* **2021**, *6*, 10348–10354.

[12] R. Nandan, N. Takamori, K. Higashimine, R. Badam, N. Matsumi, *J. Mater. Chem. A* **2022**, *10*, 15960–15974.

**Table S2.** Reversible specific capacities (based on both the anode and cathode masses) of pristine Si||LiNi_0.8_Co_0.1_Mn_0.1_O_2_ and S3HT||LiNi_0.8_Co_0.1_Mn_0.1_O_2_ full cells measured at various C rates.

| Current rate (C) | Pristine Si\|\|LiNi_0.8_Co_0.1_Mn_0.1_O_2_ | S3HT\|\|LiNi_0.8_Co_0.1_Mn_0.1_O_2_ |
| --- | --- | --- |
|  | Reversible capacity (mAh g^–1^) | Reversible capacity (mAh g^–1^) |
| 0.1 | 151 | 182 |
| 0.2 | 144 | 176 |
| 0.5 | 123 | 166 |
| 1.0 | 88 | 155 |
| High rate retention* | 58% | 85% |

* a comparison between reversible capacities at 1.0 and 0.1 C.


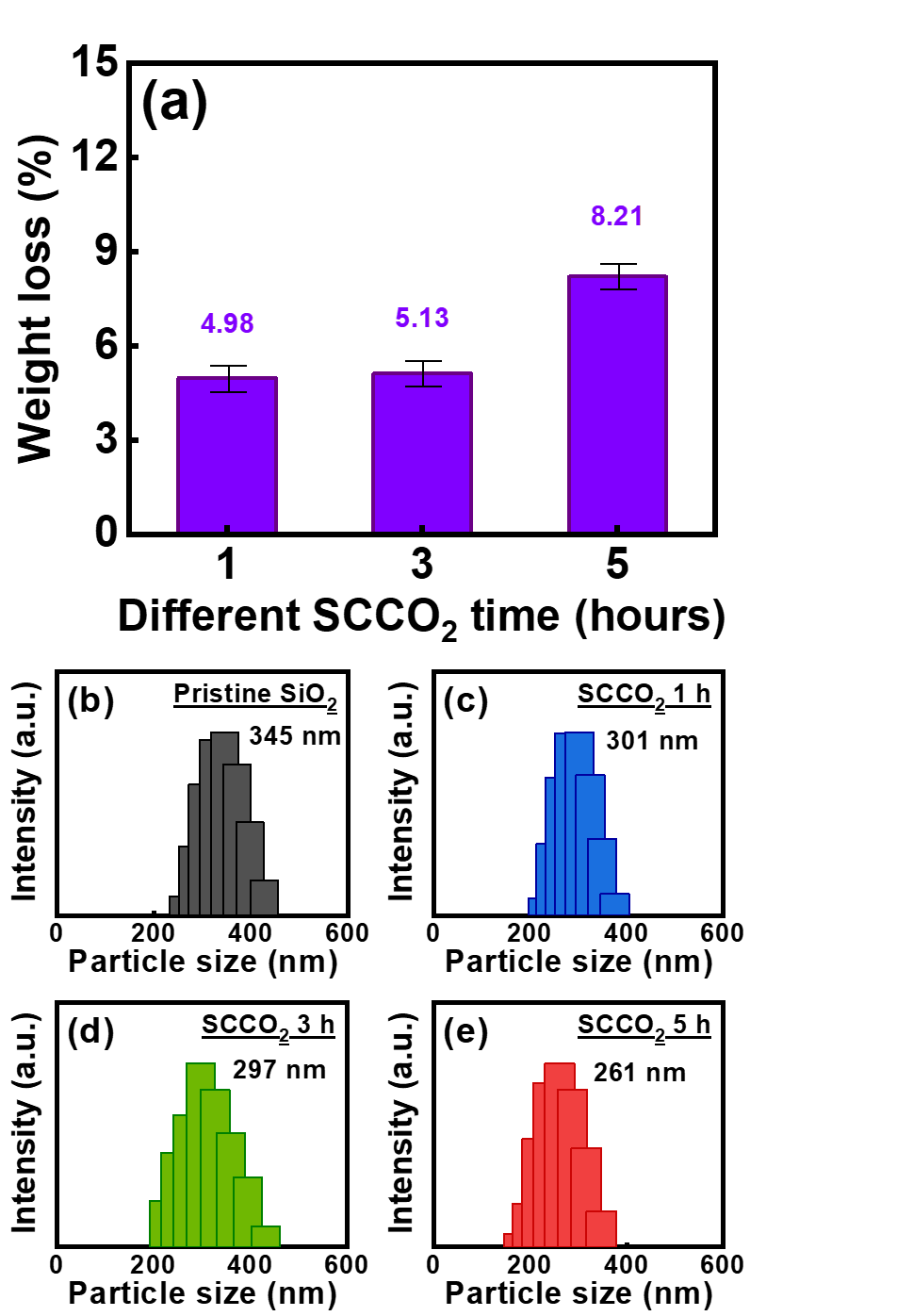


**Figure S1.** (a) Weight loss of commercial SiO_2_ powder after immersion in SCCO_2_ fluid for various times. Particle size of commercial SiO_2_ powder after immersion in SCCO_2_ fluid for (b) 0, (c) 1, (d) 3, and (e) 5 h.


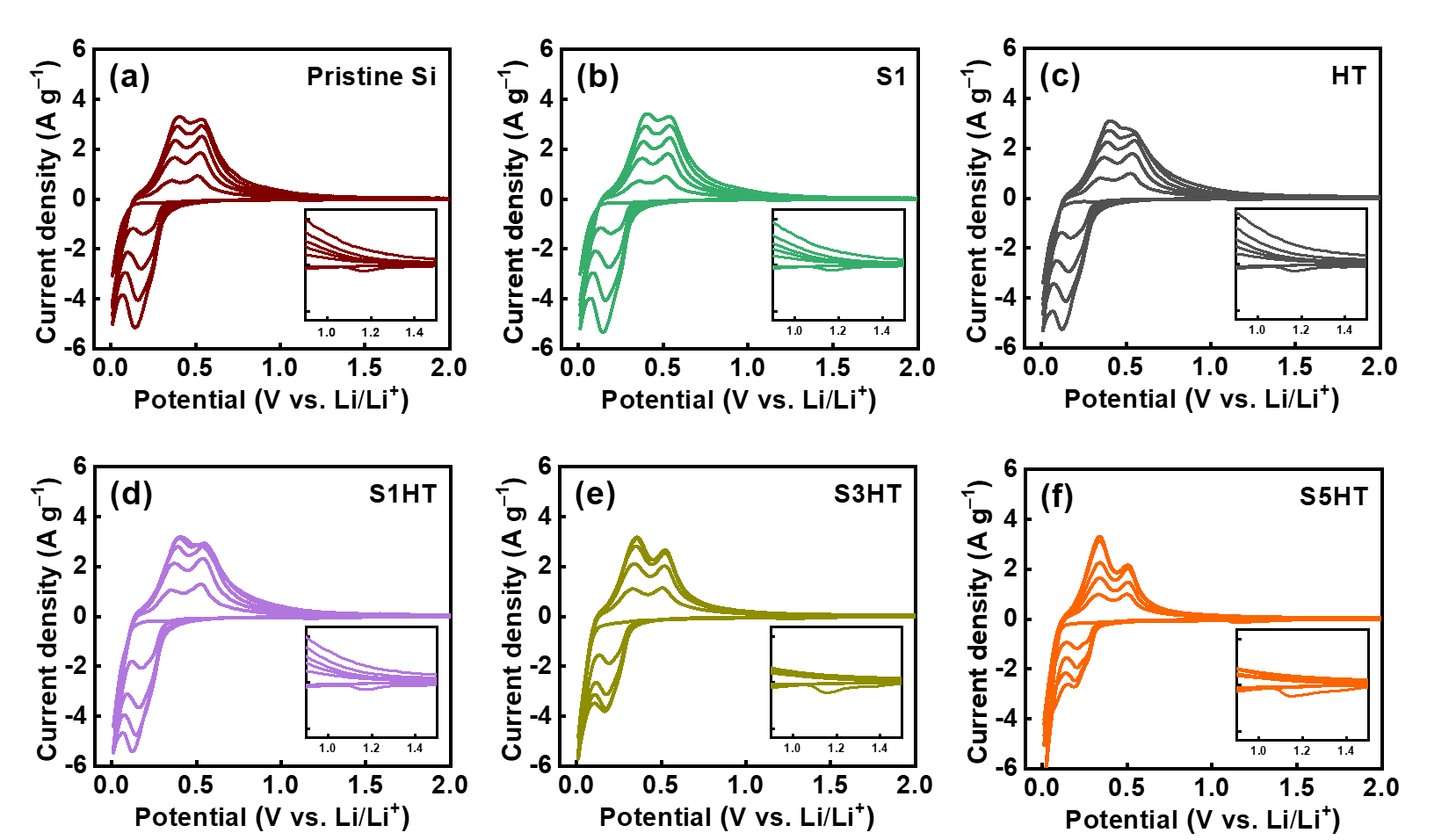


**Figure S2.** CV curves of (a) pristine Si, (b) S1, (c) HT, (d) S1HT, (e) S3HT, and (f) S5HT electrodes recorded with potential scan rate of 0.1 mV s^–1^ for 5 cycles.


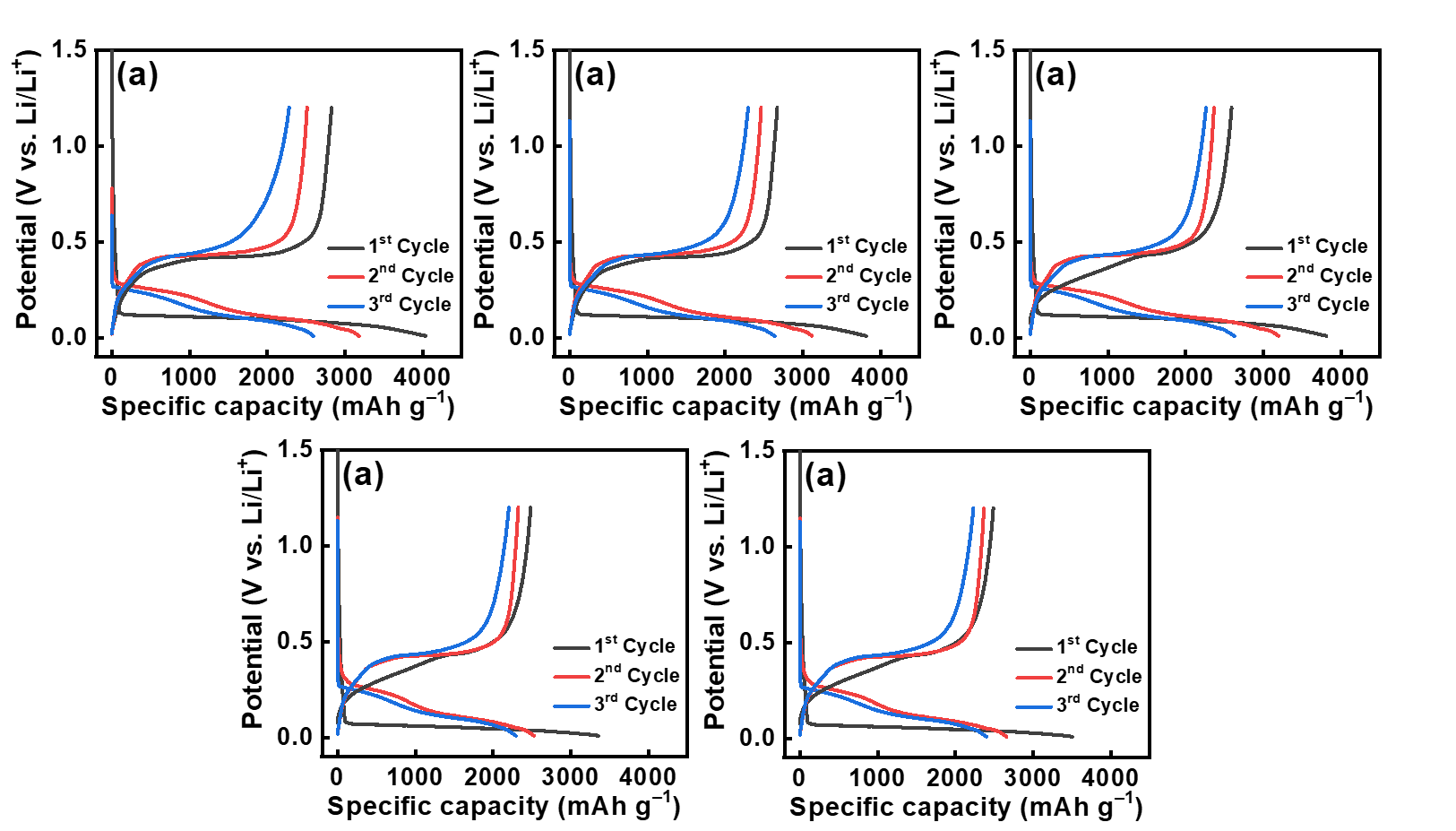


**Figure S3.** Initial three charge-discharge curves of (a) pristine Si, (b) S1, (c) HT, (d) S1HT, and (e) S5HT electrodes measured at 0.2 A g^−1^.


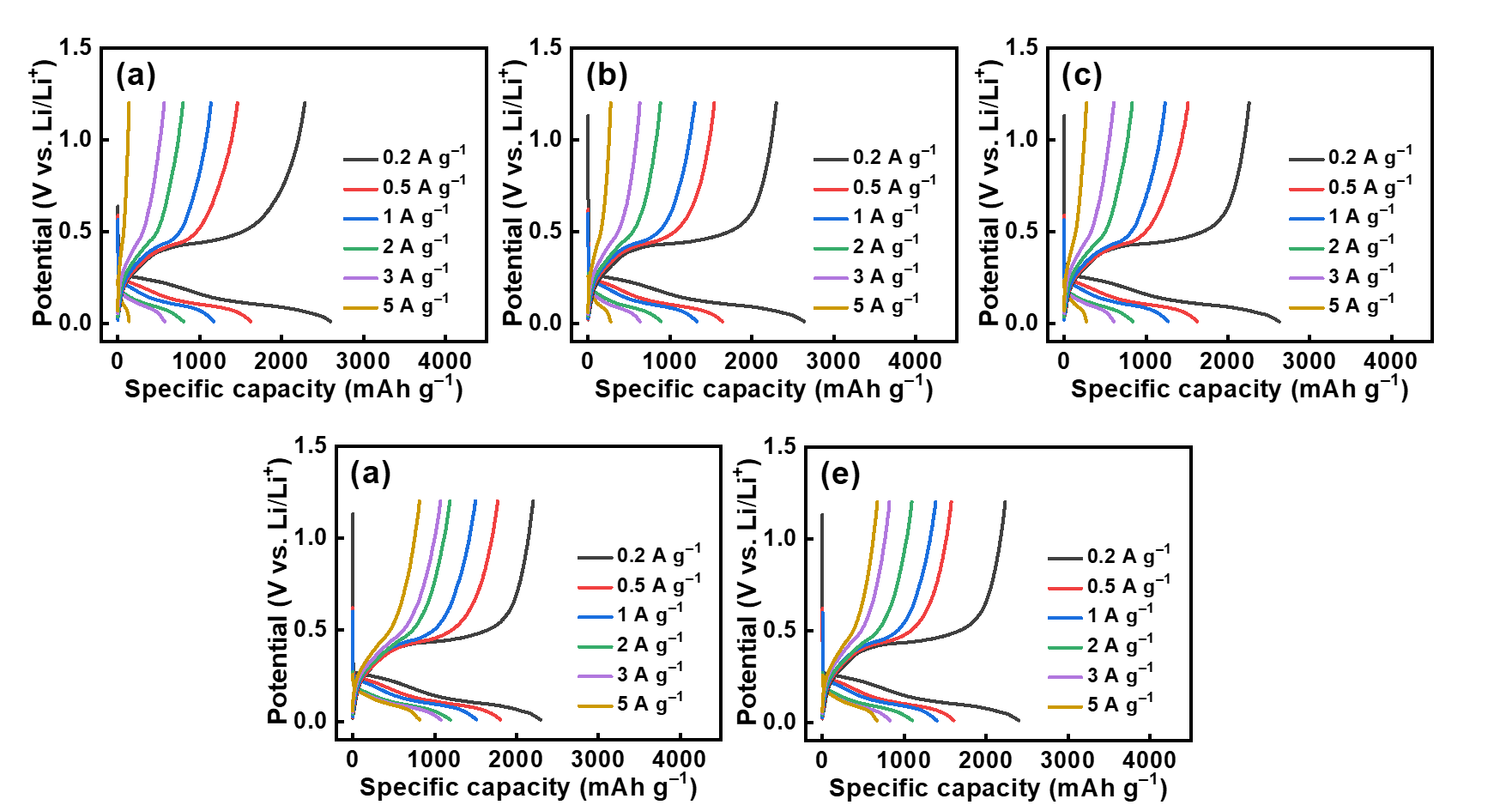


**Figure S4.** Charge-discharge curves of (a) pristine Si, (b) S1, (c) HT, (d) S1HT, and (e) S5HT electrodes measured at various current rates after two conditioning cycles.


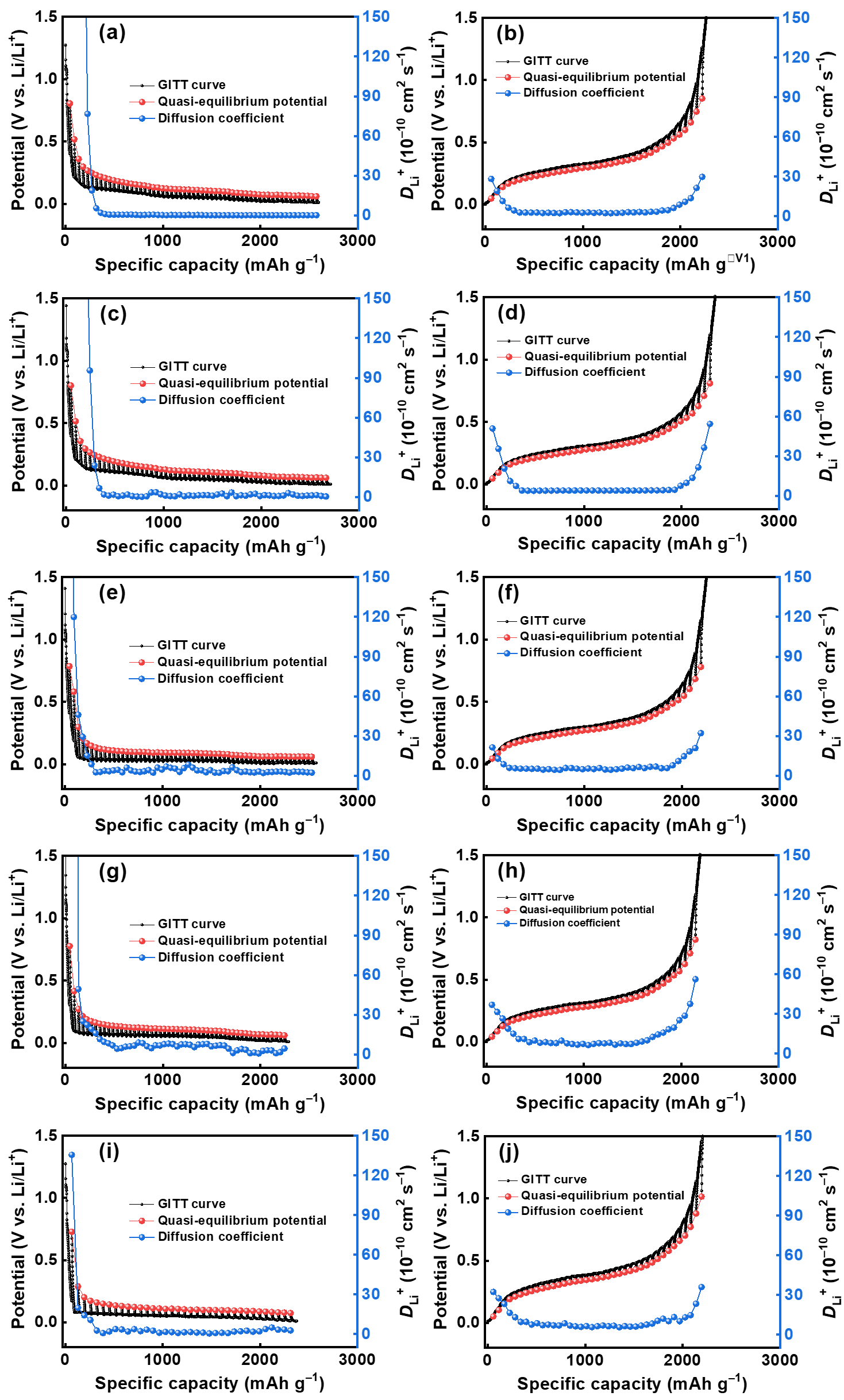


**Figure S5**. GITT measurements of (a,b) pristine Si, (c,d) S1, (e,f) HT, (g,h) S1HT, and (i,j) S5HT electrodes during lithiation and delithiation processes.

**Figure S6**. Weight loss and particle size reduction data for pristine Si and S3HT samples after immersion in 25 mM HF aqueous solution at 25 °C for 1 h.


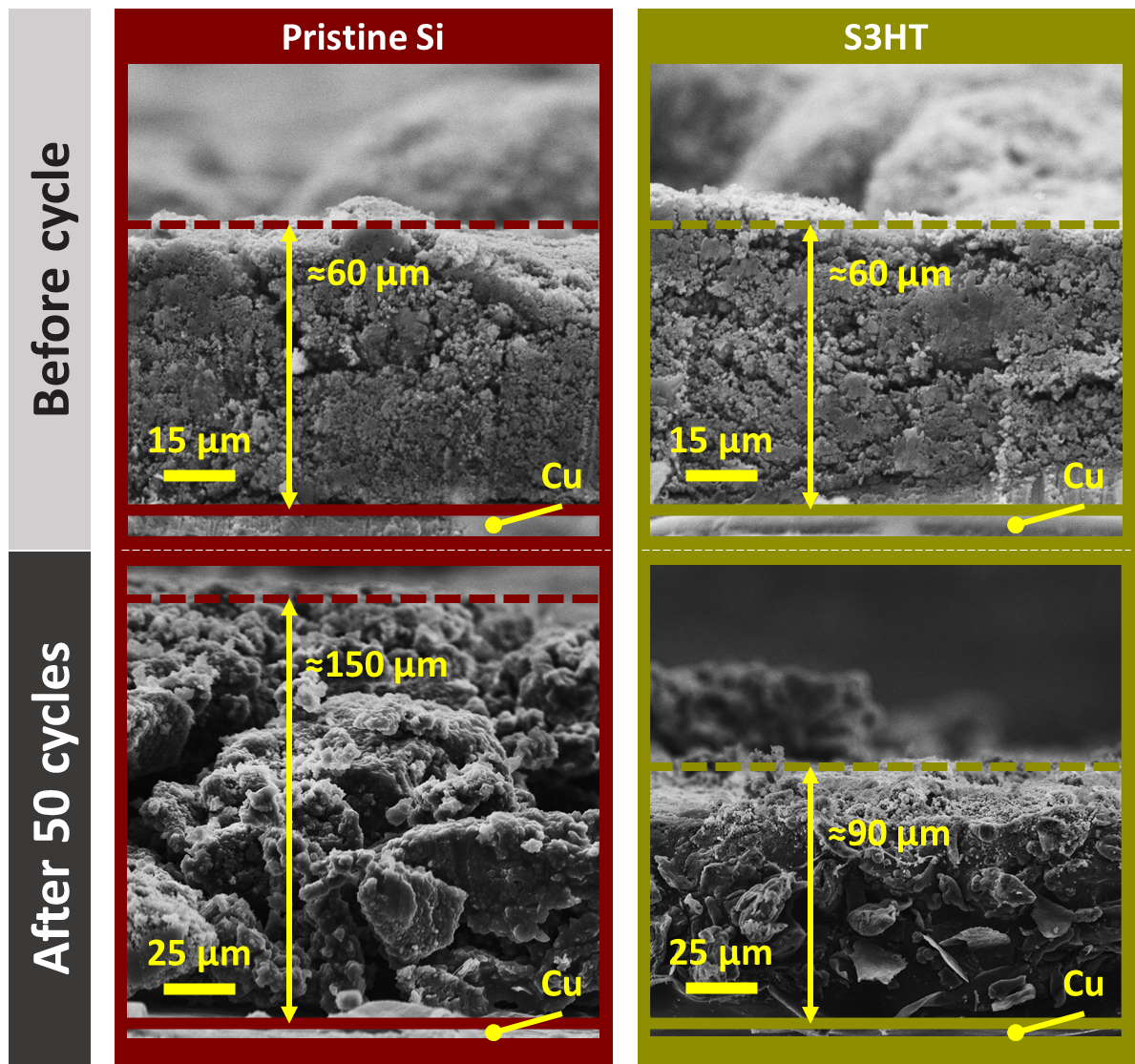


**Figure S7**. Cross-section SEM images of pristine Si and S3HT electrodes before and after 50 charge-discharge cycles.

**
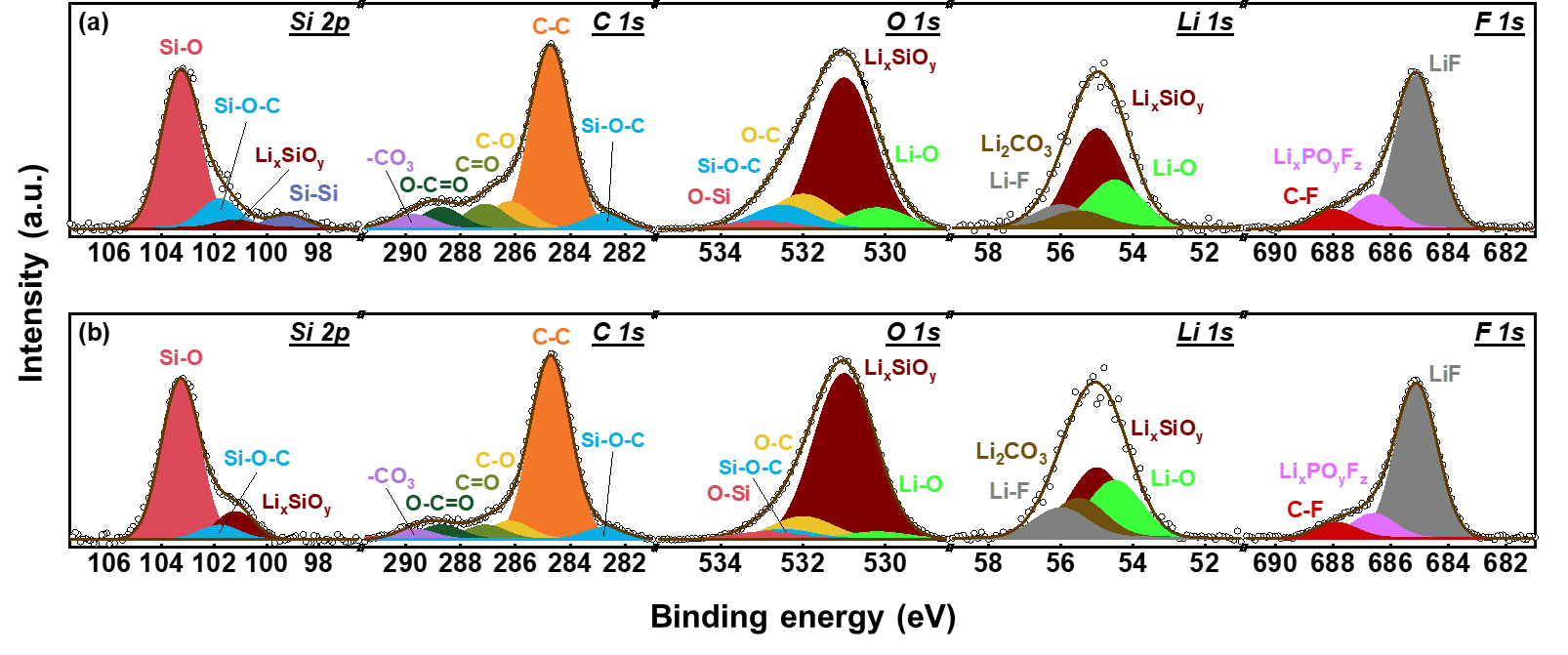
**

**Figure S8**. XPS analysis data of S3HT electrode (a) after two conditioning cycles and (b) after 300 charge-discharge cycles.
